# Proper use of antibiotics: situation of linezolid at the intensive care unit of the Tunisian Military Hospital

**DOI:** 10.11604/pamj.2016.25.196.9476

**Published:** 2016-11-28

**Authors:** Louhichi Safa, Neffati Afif, Hajjej Zied, Dridi Mehdi, Yousfi Mohamed Ali

**Affiliations:** 1 Pharmaceutical Sciences Department, Faculty of Pharmacy of Monastir, Montasir, Tunisia; 2 Pharmacy Department, Tunisian Military Hospital, Tunis, Tunisia; 3 Department of Critical Care Medicine and Anesthesiology, Tunisian Military Hospital, Tunis, Tunisie

**Keywords:** Linezolid, antibiotic use, hospital, methicillin-resistant staphylococcus aureus, antimicrobial stewardship

## Abstract

Linezolid was introduced in clinical practice in the early 2000s. It was considered to be an ideal reserve drug for treatment of vancomycin-resistant Enterococcus spp. (VRE) and vancomycin-resistant Staphylococcus aureus (VRSA). The aim of our study was to describe and evaluate the use of linezolid in clinical practice at the intensive care unit (ICU) of the Tunisian military hospital. This is a thirty-month retrospective study including patients treated with linezolid at the ICU of the Tunisian military hospital. Data collection was realized using the patients’ medical files and prescriptions. A pharmacist conducted an extended medication history and checked if an advice from an infectious disease-physician and a microbiological documentation were requested. A total of 80 patients were included. Forty-one per cent of indications were outside the Marketing Authorization (MA) criteria, and were mainly sepsis and postoperative mediastinitis (32% and 4% of total prescriptions, respectively). This antibiotic was used as a first-line therapy in 58% of cases. The advice from an infectious-disease physician was requested for 33% of prescriptions. Only 20% of infections were documented microbiologically, of which 35% were caused by methicillin resistant coagulase-negative Staphylococcus. Linezolid is an interesting therapeutic alternative in case of infections due to multi-resistant bacteria and/or complex clinical situations. Therefore, its prescription must be rationalized in order to slow down the emergence of resistance to this antibiotic. The high frequency of its use outside the MA criteria shows the importance of carrying out more clinical trials to evaluate its effectiveness and safety for new indications.

## Introduction

Linezolid is a synthetic oxazolidinone. It is an antimicrobial agent that has good activity against most medically important Gram-positive microorganisms resistant to methicillin and vancomycin. The Marketing Authorization (MA) allows linezolid for skin and soft tissue infections in addition to nosocomial and community-acquired pneumonia caused by sensitive Gram-positive bacteria. Given the need to evaluate antibiotics use and clinical practices, the prescription of linezolid at the Tunisian military hospital, one of the largest Tunisian hospitals, was investigated. The aims of our study were to judge the degree of conformity of linezolid prescriptions with the MA criteria, to evaluate the circumstances when this antibiotic was used and to specify its place in therapeutic strategy.

## Methods

It is a thirty-month retrospective study (from January 1^st^, 2013 to June 30th 2015). It was carried out in the intensive care unit (ICU) of the Tunisian military hospital. This is a 622-bed hospital. Although the intensive care unit contains only 20 beds (3%), it has been found that it is the biggest consumer of linezolid. During the study’s period, this unit consumed 81% of linezolid pockets (1409 among 1742 pockets). Treatment with linezolid in this period was the only inclusion criterion. There were no exclusion criteria. Data were collected using the patients’ medical files in addition to registered prescriptions collected by the pharmacy’s software. These data were about: demographic profile, indications for linezolid prescription, antibiotic therapeutic strategy, duration of treatment, advice from an infectious-disease physician and microbiological documentation of prescriptions. Statistical analysis was performed using Microsoft Excel 2007 software.

## Results

The total number of patients included in this work was 80. The majority of them were men (*sex-ratio (M/F*) = 2.5). Mean age was 60 (range 19-87). The indications for linezolid are summarized in Table 1. Forty-one per cent of prescriptions were outside the MA criteria, and were mainly sepsis and postoperative mediastinitis (32% and 4% of total prescriptions, respectively). Linezolid therapy was prescribed as a first-line treatment in 46 cases (58%). It was used as second-line (42%) after the prescription of glycopeptides in 15% of cases only. This antibiotic was prescribed as a monotherapy (45%) or in association with another antibiotic (55%), including 20 bitherapies (45%) and 24 multitherapies (55%). Antibiotics that were most co-administrated with linezolid included imipenem/cilastatin and colistin (80% and 39% of cases, respectively). The main reasons for prescribing linezolid include: inefficacy of other antibiotics (31%) and the alteration of renal function (9%). For the rest of patients (60%), the reason why linezolid was used wasn’t mentioned.

**Table 1:** Indications for linezolid

Indication	All patients (n = 80)
Pneumonia	32 (40)
Skin and soft tissue infections	15 (19)
Sepsis^+^	26 (32)
Postoperative mediastinitis^+^	3 (4)
Meningitis^+^	2 (3)
Endocarditis^+^	1 (1)
Other infections^+^	1 (1)

Data are n (%) of patients

+ indication outside the MA

The advice from an infectious-disease physician was requested for 33% of prescriptions. In 80% of cases, there were no microbiological documentation when linezolid was prescribed. The microorganisms isolated are mentioned in Table 2. In all patients, the usual dose (600mg twice daily) was respected. The average duration of treatment was 7 days (from 1 to 20 days). In 26% of cases, linezolid was given for less than 72 hours and it was replaced by another antibiotic based on culture and sensitivity results. All patients received linezolid parenterally. Oral switch was mentioned in 3 cases only (4%). Forty and one patients (51%) had a favorable evolution of their infection under linezolid.

**Table 2:** Microorganisms isolated from patients treated with linezolid

Microorganism isolated	Total of patients (n = 80)
MRSA	4(23)
MR-CNS	6(35)
MS-CNS	3(18)
*Enterococcus sp*	2(12)
*Streptococcus pneumoniae*	1(6)
MSSA	1(6)
Not documented	64 (80)

- Data are n (%) of patients - MRSA: methicillin resistant *Staphylococcus aureus*, MR-CNS: methicillin resistant coagulase negative *Staphylococcus*, MS-CNS: methicillin sensitive coagulase negative *staphylococcus*, MSSA: methicillin sensitive *Staphylococcus aureus*.

## Discussion

The results of this study show the significant contribution of linezolid in the treatment of some complex infections associated with the presence of multi-resistant bacteria. This antimicrobial agent has an average alveolar diffusion of 97%. Its serum and alveolar concentration exceeds twice the minimum inhibitory concentration (MIC) of *staphylococcus aureus* (4mg / L) [1]. This finding proves that linezolid is an interesting alternative for the treatment of pneumonia. However, a meta-analysis and a systematic literature review showed that the efficacy and safety profiles of linezolid and vancomycin in the treatment of nosocomial pneumonia are similar [2].

Another study has shown that linezolid has superiority over vancomycin for this indication [3]. So, we conclude that the optimal treatment of nosocomial pneumonia caused by Gram-positive bacteria remains controversial. Linezolid turned out to be more effective than glycopeptides for the treatment of complicated skin and soft tissue infections due to Gram-positive bacteria [4]. In addition, the prescription of linezolid instead of vancomycin to treat these infections in patients with peripheral vascular disease and / or diabetic shortened the duration of treatment (12.9 +/- 7.9 days for linezolid Vs 16.4 +/- 8.3 days for vancomycin, p <0.001) and the hospitalization’s period (17.9 +/- 13.6 days 22.6 for linezolid vs. 22.6 +/- 13.6 days vancomycin, p <0.001) [5]. But, it should be noted that tedizolid, a new oxazolidinone, showed a spectacular efficacy and safety for the treatment of skin and soft tissue infections suspected or documented to be caused by Gram-positive bacteria in adults. Phase III clinical trials have shown that this antibiotic, administrated at a dose of 200 mg / day for 6 days had an equal effect to that of linezolid (600 *2 mg /day for 10 days). This molecule can be administered per os. It has almost the same side effects as linezolid and a less potential for drug interactions [6].

In our study, 41% of prescriptions were outside the MA criteria, but 97% of these were justified with regard to current data from literature [7–15]. Sepsis microbiologically documented to be caused by Gram-positive bacteria (5/26) are mainly due to two germs: Staphylococcus aureus and coagulase-negative Staphylococcus (3 and 2 cases respectively). According to a multicenter epidemiological study conducted in the United States, it has been shown that these germs are the most implicated in nosocomial septicemia (20% and 31% of isolates, respectively) [16]. It should be noted that linezolid was used as a second-line therapy for the treatment of sepsis. In fact, this antimicrobial agent and vancomycin have almost the same effectiveness against Staphylococcus genus [7, 8]. Moreover, the resistance of this genus to vancomycin is currently less described compared to Enterococcus genus which has an increasingly diminished sensitivity to vancomycin, daptomycin and even linezolid [9–12]. Mediastinitis is a common complication of cardiovascular surgeries. This infection can be caused by MRSA and is associated with high morbidity and mortality [17]. Tsuji et al showed that linezolid’s concentration in the mediastinum and pleural space varies as in the serum and that the concentration in the mediastinum is the same as or greater than that in the serum [13]. Thus, linezolid is an interesting therapeutic alternative in mediastinitis due to MRSA.

In meningitis and endocarditis, this antibiotic was used as a bacteriostatic agent [14, 15]. The duration of treatment didn’t exceed 20 days unlike other publications where treatment duration was longer [18]. For the treatment of endocarditis, a retrospective study was performed on 33 patients with endocarditis caused by MRSA or GISA (glycopeptide-intermediate Staphylococcus aureus). The rate of successful treatment with linezolid hasn’t exceeded 65% (63.6%). For infections of the central nervous system, a review of published cases identified 42 patients treated with linezolid with a cure rate of 90.5% [19, 20]. Given the lack of robust clinical studies that advocate the prescription of linezolid in these two indications, it should be used with caution in cases where another therapeutic alternative is not available.

One patient was treated with linezolid for an indication which is not described in the literature: an inflammatory syndrome. In this context, a recent study showed that linezolid attenuates the excessive inflammatory response in pneumonia caused by Gram-positive bacteria by several mechanisms [21]. But this observation doesn’t justify the use of this antibiotic without specifying the indication and in the absence of microbiological documentation. In the study of Guillard and al, 45% of prescriptions were outside the MA criteria which is almost of product to the results of our study [22]. However, other studies have shown different results such as the study of Aubin and al at the University Hospital of Nantes (61.1%) and of Angers (71.5%) [23]. These studies have also shown a larger variety of indications. The association of linezolid with other antibiotics has concerned no documented infections where many microorganisms were suspected and vital prognosis was threatened.

The main reasons for using linezolid were almost similar to those of the study of Aubin and al but we noticed that this French study had tried to show the advantages of linezolid’s oral form. In fact, 18% of the reasons for prescription (hospital discharge with need to continue the treatment and the impossibility of inserting a venous line) are in relation with the benefits of this form. In addition, a shift to oral administration was mentioned in 22% of patients in this study versus 4% in ours. It is explained by the fact that our study was carried out at the ICU where the majority of patients were intubated and ventilated so the use of oral route was impossible. Despite its advantages, the oral form of linezolid is poorly exploited by clinical services at the Tunisian military hospital [24]. Our study showed that 80% of prescriptions were not documented. This percentage is almost equal to twice that of the Aubin and al study (47%). The most isolated germ in our study is Methicillin-resistant coagulase-negative *Staphylococcus* (35%) versus 12% in the aforementioned study. This result proves that bacterial ecology differs from one hospital to another and from one country to another.

The advice from an infectious-disease physician was requested for 33% of prescriptions versus 65% in the study of Aubin and al [18]. It should be noted that these cases (26) correspond to linezolid prescriptions received during the first six months of 2015. This is the period from which the Tunisian military hospital started using the validated prescriptions. These prescriptions require approval by an infectious-disease physician selected from a predetermined list in order to provide the antibiotic. Applied in Europe for more than five years, the program ´Antimicrobial stewardship´ has just started to be applied at the Tunisian military hospital [25]. This hospital is the first to apply this strategy in Tunisia. According to the results of a recent international study, only 13% of African countries are implementing this program versus 65% in Europe [26]. This is due mainly to a lack of funding and qualified staff (29% of cases).

Finally, we should mention that this study has limitations. The most important is its retrospective nature. Some data were not found in the patients’ medical files such as the reasons for linezolid’s prescription. Similarly, some factors were underestimated as the opinion of an infectious-disease physician in case of non-documented infection and the evolution of the patient´s situation.

## Conclusion

No case of resistance to linezolid has been reported since the beginning of its prescription at the Tunisian military hospital. But it should be noted that an increase of Gram-positive bacteria’ resistance to this antibiotic has been noted in several countries. This requires rationalizing its use. The important number of prescriptions outside the MA criteria should encourage researchers to carry out more clinical trials to prove the authenticity of its use for these indications. The oral form of this antibiotic need to be better exploited when the patient's situation permits. In fact, it is an effective solution when the use of an intravenous line is impossible or to shorten the duration of hospitalization. As part of the implementation of the 'Antimicrobial Stewardship' strategy, an effective collaboration between clinicians, microbiologists and pharmacists will guarantee the proper use of this antimicrobial agent. Subsequent studies will show the impact of this strategy on antibiotic use and on the management of bacterial resistance at the Tunisian military hospital.
